# Human C1orf27 protein interacts with α_2A_-adrenergic receptor and regulates its anterograde transport

**DOI:** 10.1016/j.jbc.2022.102021

**Published:** 2022-05-10

**Authors:** Xin Xu, Guangyu Wu

**Affiliations:** Department of Pharmacology and Toxicology, Medical College of Georgia, Augusta University, Augusta, Georgia, USA

**Keywords:** α_2_-adrenergic receptor, C1orf27, ER, export, Golgi, G protein-coupled receptor, maturation, ODR4, signaling, trafficking, α_2A_-AR, α_2A_-adrenergic receptor, β_2_-AR, β_2_-adrenergic receptor, BRET, bioluminescence resonance energy transfer, CHX, cycloheximide, CT, C terminus, D2R, dopamine D2 receptor, DMEM, Dulbecco's modified Eagle's medium, EGF, epidermal growth factor, EGFR, EGF receptor, ER, endoplasmic reticulum, ERGIC, ER–Golgi intermediate complex, ERK1/2, extracellular signal–regulated kinase 1 and 2, FBS, fetal bovine serum, GPCR, G protein-coupled receptor, GFP/RFP, green/red fluorescent protein, GST, glutathione S-transferase, HA, hemagglutinin, HEK293, human embryonic kidney 293, ICL3, third intracellular loop, KO, knockout, NT, N terminus, ODR4, odorant response abnormal 4, PBS, phosphate-buffered saline, PM, plasma membrane, RUSH, retention using the selective hooks, SBP, streptavidin binding peptide, sgRNA, single guide RNA, siRNA, small interfering RNA

## Abstract

The molecular mechanisms underlying the anterograde surface transport of G protein–coupled receptors (GPCRs) after their synthesis in the endoplasmic reticulum (ER) are not well defined. In *C. elegans,* odorant response abnormal 4 has been implicated in the delivery of olfactory GPCRs to the cilia of chemosensory neurons. However, the function and regulation of its human homolog, C1orf27, in GPCR transport or in general membrane trafficking remain unknown. Here, we demonstrate that siRNA-mediated knockdown of C1orf27 markedly impedes the ER-to-Golgi export kinetics of newly synthesized α_2A_-adrenergic receptor (α_2A_-AR), a prototypic GPCR, with the half-time being prolonged by more than 65%, in mammalian cells in retention using the selective hooks assays. Using modified bioluminescence resonance energy transfer assays and ELISAs, we also show that C1orf27 knockdown significantly inhibits the surface transport of α_2A_-AR. Similarly, C1orf27 knockout by CRISPR-Cas9 markedly suppresses the ER–Golgi-surface transport of α_2A_-AR. In addition, we demonstrate that C1orf27 depletion attenuates the export of β_2_-AR and dopamine D2 receptor but not of epidermal growth factor receptor. We further show that C1orf27 physically associates with α_2A_-AR, specifically *via* its third intracellular loop and C terminus. Taken together, these data demonstrate an important role of C1orf27 in the trafficking of nascent GPCRs from the ER to the cell surface through the Golgi and provide novel insights into the regulation of the biosynthesis and anterograde transport of the GPCR family members.

G protein–coupled receptors (GPCRs) represent the largest superfamily of cell surface signaling proteins that regulate a wide variety of cell functions under physiological and pathological conditions (1, 2). The life of GPCRs begins in the endoplasmic reticulum (ER) where they are synthesized. Once correctly folded and properly assembled, nascent receptors are able to pass the scrutiny of the ER quality control system and export from the ER through the Golgi apparatus to the cell surface where they can bind to their cognate ligands to activate specific downstream signaling molecules (3). Although the ER export and subsequent forward transport of newly synthesized GPCRs are well known to dictate the number of receptors at the functional destinations, control the magnitude and duration of receptor-elicited cellular responses, and contribute to the development of human diseases (4, 5, 6, 7, 8, 9, 10, 11, 12), it remains not well understood how their targeted anterograde delivery is achieved.

α_2_-Adrenergic receptors (α_2_-ARs) are prototypic members of the GPCR family that have three subtypes, α_2A_-AR, α_2B_-AR, and α_2C_-AR, all of which play important roles in regulating the sympathetic nervous system, both peripherally and centrally. All α_2_-AR subtypes couple to the Gi/Go family of G proteins and regulate the activation of adenylyl cyclases, Ca^2+^ channels, and mitogen-activated protein kinases (13, 14, 15, 16). A structural feature of α_2_-ARs is that they have quite large third intracellular loop (ICL3) and relatively short C terminus (CT), both are important intracellular domains, mediating receptor interaction with G proteins, arrestins, protein kinases, and other molecules involved in regulation of signal initiation and termination, phosphorylation, and trafficking (17, 18, 19, 20, 21, 22).

Odorant response abnormal 4 (ODR4) gene was identified in chemotaxis-defective screens in *C. elegans* (23) and its protein product was later found to be expressed exclusively on intracellular membranes in chemosensory neurons. ODR4 is predicted to have a C-terminal transmembrane domain, structurally similar to syntaxins, a family of proteins involved in vesicle-mediated transport (24). The function of ODR4 has been demonstrated to enhance the surface delivery of some olfactory receptors (ORs), such as *C. elegans* ODR10 in specialized chemosensory neurons (24, 25) and rat U131 in undifferentiated olfactory-derived odorant receptor activatable (*odora)* cells and Chinese hamster ovary cells (26). ORs are specific GPCRs that are expressed in the cilia and synapses of OR neurons and are responsible for the detection of odor molecules. Although ORs are efficiently expressed at the cell surface in olfactory neurons, they are retained in intracellular compartments (*e.g.*, ER, Golgi, and endosomes) when expressed in heterologous systems.

C1orf27 is a human homolog of ODR4 and ubiquitously expressed (27). *C. elegans* ODR4 and human C1orf27 share only 22% identity and 44% similarity. Recent studies suggest that C1orf27 is involved in regulation of protein UFMylation/deUFMylation *via* interaction with ubiquitin-fold modifier 1-specific protease 2, a deUFMylation enzyme (28). However, its function in specific GPCR transport or general membrane trafficking has not been investigated. In this study, we determine the role of C1orf27 in the ER–Golgi-surface transport of α_2A_-AR and elucidate the possible underlying mechanisms in mammalian cells. We show that C1orf27 depletion significantly inhibits the ER–Golgi transport and surface expression of newly synthesized α_2A_-AR and that this function of C1orf27 is likely mediated through direct interaction with the receptor. These data demonstrate an important role of C1orf27 in α_2A_-AR trafficking along the biosynthetic pathway and reveal a novel mechanism governing the anterograde delivery of nascent GPCRs.

## Results

### Characterization of the anterograde transport kinetics of nascent α_2A_-AR

**Figure 1:**
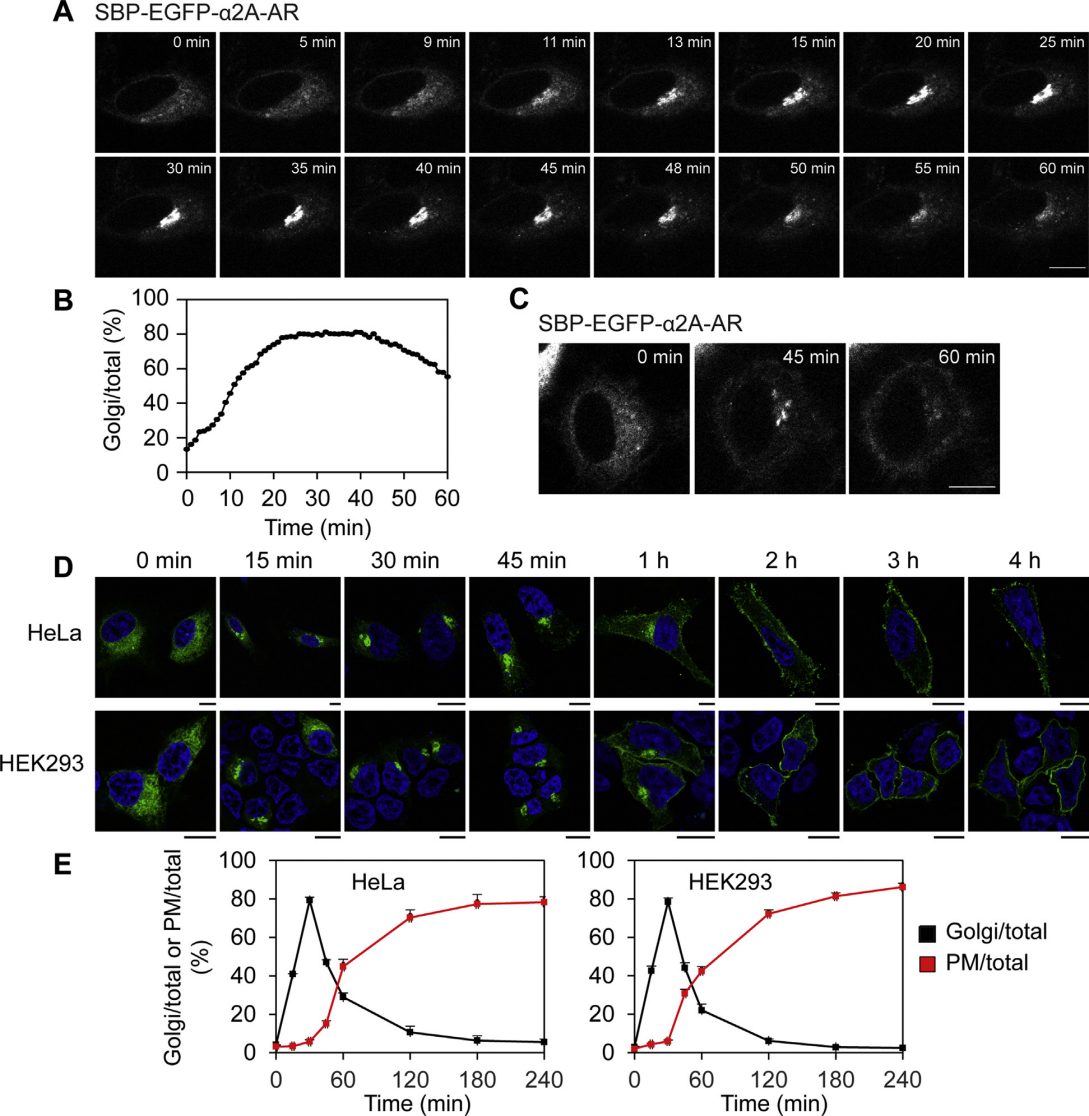
**Characterization of anterograde transport of nascent α**_**2A**_**-AR in RUSH assays**. *A*, α_2A_-AR export from the ER over time in RUSH assays in live cells. HeLa cells were transfected with Str-KDEL_SBP-EGFP-α_2A_-AR plasmids for 20 h, and the ER export of α_2A_-AR was induced by addition of biotin at 0 min. *B*, quantitative data shown in A. The data are expressed as the ratio of Golgi expression to total expression of α_2A_-AR. *C*, images showing the export of α_2A_-AR from the ER to the surface. *D*, the ER–Golgi–PM transport of α_2A_-AR in RUSH assays in fixed cells. HeLa or HEK293 cells were transfected with Str-KDEL_SBP-EGFP-α_2A_-AR plasmids for 20 h and fixed at different time after addition of biotin. *E*, quantitative data shown in D. The quantitative data shown in *E* are the Golgi/total or PM/total ratio and expressed as mean ± SE (n = 11–42 cells in five separate experiments). Scale bars, 10 μm. α2A-AR, α2A-adrenergic receptor; ER, endoplasmic reticulum; PM, plasma membrane; RUSH, retention using the selective hooks; SBP, streptavidin binding peptide.

To investigate the possible function of C1orf27 in the anterograde export of GPCRs in mammalian cells, we first used the retention using the selective hooks (RUSH) assays (29) to characterize the transport properties of newly synthesized α_2A_-AR in HeLa and HEK293 cells. In RUSH assays, the cargo of interest is fused to green fluorescent protein (GFP) and a streptavidin-binding peptide (SBP) at its N terminus (NT) and the ER retention signal KDEL fused to streptavidin (Str-KDEL) is used as a hook to prevent the export of nascent cargo from the ER *via* streptavidin–SBP interaction. The ER export of cargo molecules is synchronized after incubation with biotin that binds streptavidin and thus disrupts SBP–streptavidin interaction. We first generated the RUSH construct Str-KDEL_SBP-EGFP-α_2A_-AR and tested the RUSH system in the transport of α_2A_-AR from the ER through the Golgi apparatus to the cell surface in live HeLa cells. α_2A_-AR was almost exclusively accumulated in the ER in the absence of biotin (Fig. 1*A*, 0 min) and incubation with biotin induced α_2A_-AR export from the ER to the Golgi and the cell surface. After addition of biotin for about 10 min, the cargo α_2A_-AR was clearly concentrated at the Golgi, and the strongest Golgi expression was observed at 25 to 40 min (Fig. 1, *A* and *B*). After 45 min of biotin induction, α_2A_-AR expression at the Golgi began to decline, indicative of transport to the cell surface (Fig. 1, *A* and *B*). Although α_2A_-AR surface expression was visible after 1 h of biotin induction in some cells (Fig. 1*C*), robust receptor expression at the surface was not always easily detected, likely due to the mobility of live cells and low abundance of the receptor in cells chosen to be studied.

We then compared the transport kinetics of α_2A_-AR in HEK293 and HeLa cells after the cells were transfected with the RUSH plasmid and fixed at different time of biotin induction. Similar to the results observed in live cells, α_2A_-AR transport to the Golgi was obvious at 15 min after biotin induction and reached the maximum at 30 to 45 min (Fig. 1*D*). α_2A_-AR transport to the Golgi after release from the ER was confirmed by co-localization with the Golgi markers pmTurquoise2-Golgi and β1,4-galactosyltransferase 1 (GalT) (Fig. S1). α_2A_-AR expression at the surface was clearly detected in fixed cells which was accompanied by the reduction in the Golgi expression after 1 h of biotin addition, and the surface expression reached the plateau after 2 h induction (Fig. 1, *D* and *E*). In addition, the ER–Golgi transport of α_2A_-AR over time in HEK293 and HeLa cells was very much the same (Fig. 1, *D* and *E*).

### siRNA-mediated knockdown of C1orf27 impedes the ER-to-Golgi transport of nascent α_2A_-AR

**Figure 2:**
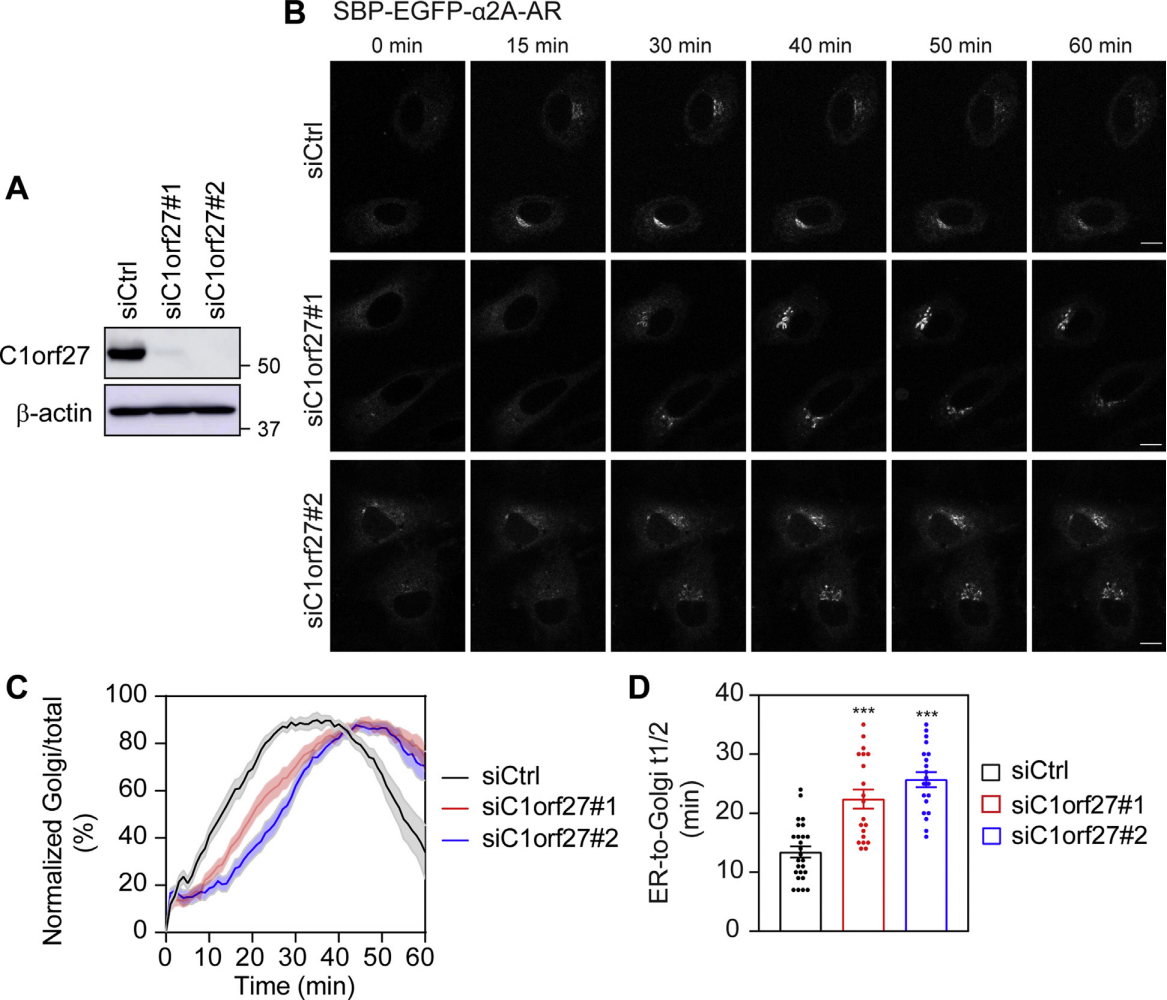
**siRNA-mediated depletion of C1orf27 impedes the ER-to-Golgi transport kinetics of nascent α**_**2A**_**-AR**. *A*, Western blot analysis of siRNA-mediated knockdown of endogenous C1orf27. *B*, effect of C1orf27 knockdown by siRNA on the ER–Golgi transport kinetics of α_2A_-AR. HeLa cells were transfected with Str-KDEL_SBP-EGFP-α_2A_-AR plasmids together with control or C1orf27 siRNA. α_2A_-AR transport from the ER was induced after addition of biotin at 0 min. *C*, quantitative data showing the normalized Golgi/total expression of α_2A_-AR over time in control or C1orf27 siRNA-transfected cells. After addition of biotin, images were captured at an interval of 1 min. The Golgi/total ratio at each time was normalized to the highest ratio after subtraction from the ratio at time 0 in individual cells. *D*, the half time of ER–Golgi transport of α_2A_-AR in control and C1orf27 siRNA-transfected cells. The quantitative data are mean ± SE (n = 20–26 cells in 6–10 individual experiments). ∗∗∗*p* < 0.001 *versus* control siRNA. Scale bars, 10 μm. α2A-AR, α2A-adrenergic receptor; ER, endoplasmic reticulum.

We next measured the effect of siRNA-mediated knockdown of C1orf27 on the ER–Golgi transport of α_2A_-AR by quantifying its expression at the Golgi over time. Transfection of siRNA targeting C1orf27 almost abolished the expression of C1orf27 (Fig. 2*A*). In live cell RUSH assays, similar to the results observed in cells without transfection, α_2A_-AR was strongly expressed at the Golgi after 15 min of biotin addition, and the majority of α_2A_-AR was transported to the Golgi at 30 min after biotin treatment in control siRNA-transfected cells (Fig. 2*B*), suggesting that control siRNA did not affect the transport kinetics of α_2A_-AR. In marked contrast, α_2A_-AR was not clearly transported to the Golgi until biotin incubation for 30 min, and the strongest Golgi expression was observed at about 50 min in C1orf27 siRNA-treated cells (Fig. 2*B*). Quantitative data showed that the time course curves of α_2A_-AR expression at the Golgi were shifted to the right in C1orf27 siRNA-transfected cells as compared with control cells (Fig. 2*C*) and that the half time (t_1/2_) values were much higher in two C1orf27 siRNA-transfected cells (22.4 ± 1.6 and 25.7 ± 1.2 min) than that in control siRNA-transfected cells (13.4 ± 0.9 min) (Fig. 2*D*).

**Figure 3:**
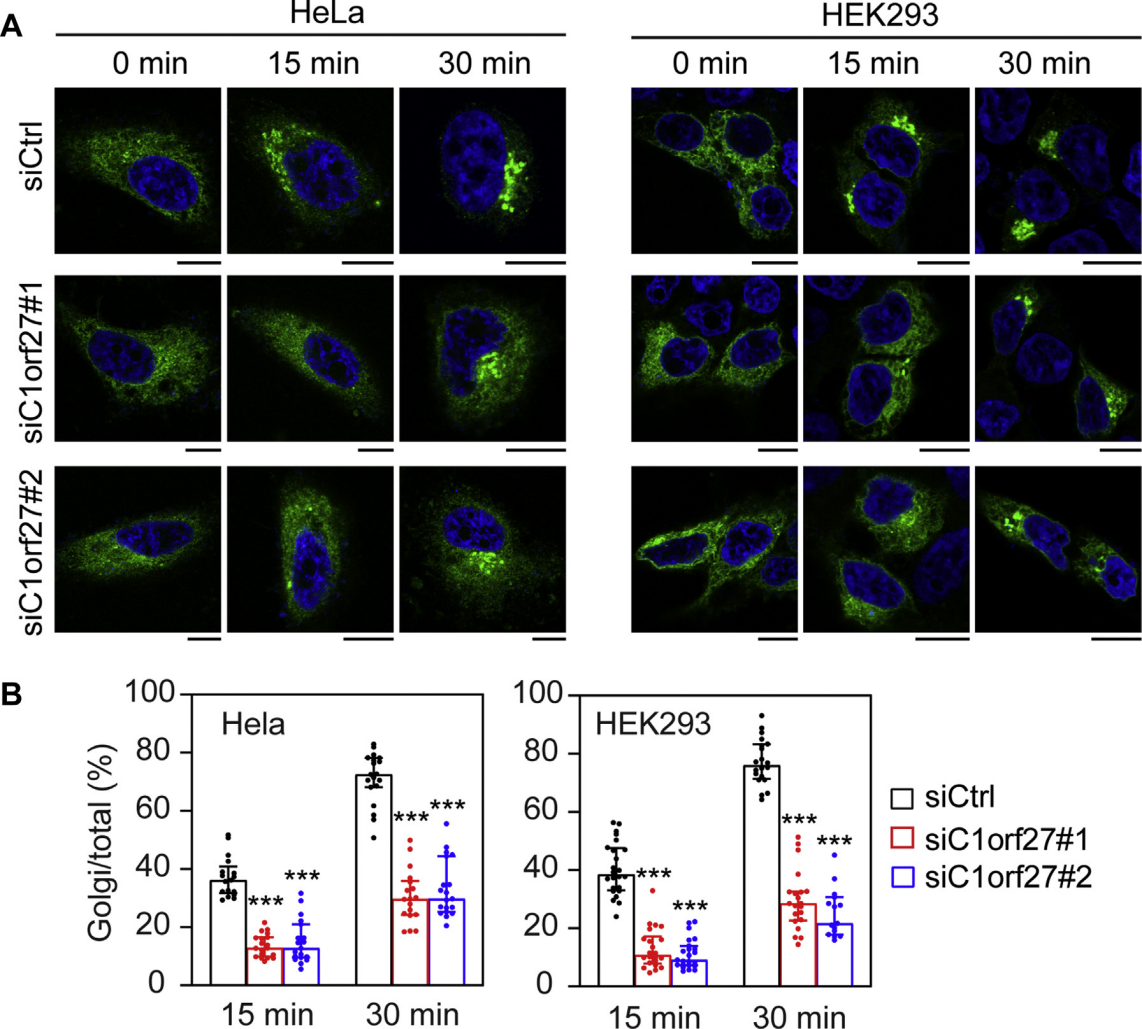
**C1orf27 depletion inhibits the transport of α**_**2A**_**-AR from the ER to the Golgi in different cell types.***A*, effect of C1orf27 knockdown by siRNA on α_2A_-AR export from the ER to the Golgi in RUSH assays in fixed cells. HeLa or HEK293 cells were transfected with Str-KDEL_SBP-EGFP-α_2A_-AR plasmids together with control or C1orf27 siRNA and fixed at 15 and 30 min after addition of biotin. *B*, quantitative data shown in A. The quantitative data are the Golgi/total ratio and expressed as mean ± SE (n = 15–24 cells in 3–4 experiments). ∗∗∗*p* < 0.001 *versus* control siRNA. Scale bars, 10 μm. α2A-AR, α2A-adrenergic receptor; ER, endoplasmic reticulum; RUSH, retention using the selective hooks.

In fixed cell RUSH assays, α_2A_-AR expression at the Golgi was significantly less in C1orf27 knockdown cells as compared with control cells at 15 and 30 min in both HEK293 and HeLa cell types (Fig. 3, *A* and *B*). Together, these data strongly demonstrate that siRNA-mediated depletion of C1orf27 remarkably slows down α_2A_-AR transport from the ER to the Golgi.

### C1orf27 depletion inhibits the surface expression and signaling of α_2A_-AR

**Figure 4:**
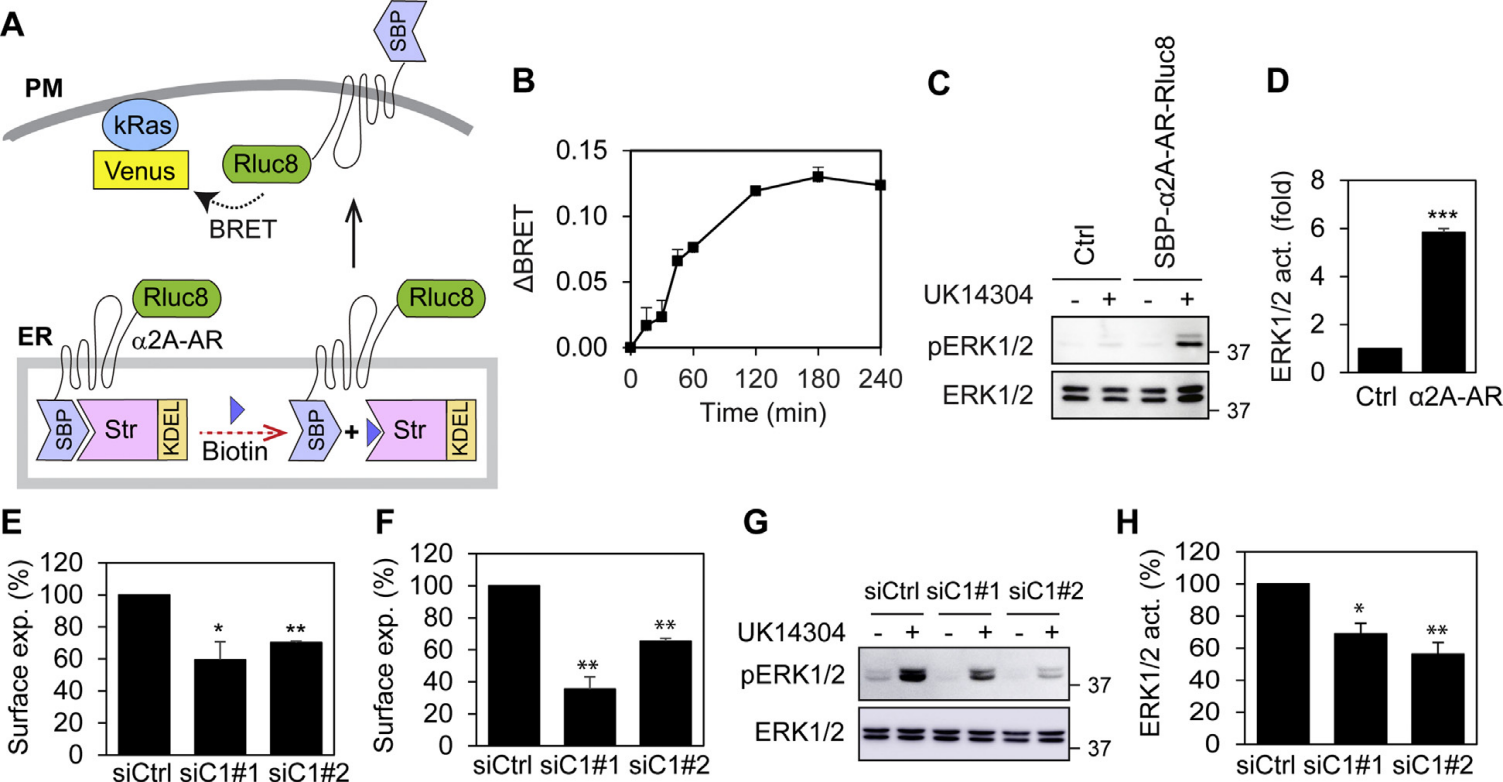
**C1orf27 depletion attenuates the surface transport and signaling of α**_**2A**_**-AR**. *A*, schematic diagram showing modified BRET assays to measure the cell surface transport of nascent α_2A_-AR after synthesis in the ER and induction with biotin using the RUSH system. *B*, the surface transport of α_2A_-AR as measured in live cell RUSH-based BRET assays. HEK293 cells were transfected with Str-KDEL_SBP-α_2A_-AR-Rluc8 and Venus-kRas for 20 h and then induced with biotin. The surface expression of α_2A_-AR was measured by BRET assays. *C*, ERK1/2 activation by Str-KDEL_SBP-α_2A_-AR-Rluc8. HEK293 cells transfected with Str-KDEL_SBP-α_2A_-AR-Rluc8 or control plasmids were treated with biotin for 2 h and then stimulated with UK14304 at 1 μM for 5 min. *D*, quantitative data shown in C. *E*, inhibition of the surface transport of α_2A_-AR by C1orf27 siRNA as measured in live cell RUSH-based BRET assays. HEK293 cells were transfected with Str-KDEL_SBP-α_2A_-AR-Rluc8 and Venus-kRas together with control or C1orf27 siRNA. After incubation with biotin for 2 h, the surface expression of α_2A_-AR was measured by BRET assays. *F*, inhibition of the surface expression of α_2A_-AR by C1orf27 siRNA as measured in ELISA. *G*, effect of C1orf27 knockdown on α_2A_-AR–mediated ERK1/2 activation. HEK293 cells were transfected with α_2A_-AR together with control or C1orf27 siRNA and stimulated with UK14304 at 1 μM for 5 min. *H*, quantitative data shown in G. The quantitative data are mean ± SE (n = 3). ∗*p* < 0.05, ∗∗*p* < 0.01 and ∗∗∗*p* < 0.001 *versus* control. α2A-AR, α2A-adrenergic receptor; BRET, bioluminescence resonance energy transfer; ER, endoplasmic reticulum; ERK1/2, extracellular signal–regulated kinase 1 and 2; PM, plasma membrane; RUSH, retention using the selective hooks.

In order to quantify the effect of C1orf27 depletion on the surface transport of α_2A_-AR, we modified bioluminescence resonance energy transfer (BRET) assays (30, 31) to measure the cell surface–receptor expression using RUSH plasmids. In this assay, Rluc8 was fused to the CT of SBP-α_2A_-AR to generate the RUSH plasmid Str-KDEL_SBP-α_2A_-AR-Rluc8, which was then transfected into HEK293 cells together with the plasma membrane (PM) marker Venus-kRas and control siRNA or individual siRNA targeting C1orf27. We first measured the time course of α_2A_-AR surface expression and found that similar to the results quantified by imaging (Fig. 1*E*), the maximal receptor expression was observed after 2 h of biotin induction (Fig. 4*B*). SBP-α_2A_-AR-Rluc8 was able to activate extracellular signal–regulated kinase 1 and 2 (ERK1/2) in response to stimulation with UK14304, an α_2_-AR agonist, suggesting that the receptor was functional (Fig. 4, *C* and *D*). The cell surface expression of α_2A_-AR was significantly attenuated in C1orf27 siRNA-transfected cells as compared with that in control siRNA-transfected cells after 2 h of biotin induction (Fig. 4*E*).

We next measured the effect of C1orf27 depletion on the surface expression of transiently expressed α_2A_-AR at steady state. In this experiment, HEK293 cells were transfected with HA-α_2A_-AR together with control or C1orf27 siRNA, and the cell surface expression of α_2A_-AR was measured in enzyme-linked immunosorbent assays (ELISA). The surface expression of α_2A_-AR was strongly reduced by more than 35% in cells transfected with C1orf27 siRNA as compared with cells transfected with control siRNA (Fig. 4*F*). These data demonstrate that in addition to the ER–Golgi transport, C1orf27 also regulates the surface expression of α_2A_-AR at steady state.

To define if C1orf27 could affect the concomitant function of α_2A_-AR, we measured the activation of ERK1/2. Consistent with the reduction in surface expression of α_2A_-AR, ERK1/2 activation after UK14304 stimulation was markedly reduced in C1orf27 knockdown cells as compared with control cells (Fig. 4, *G* and *H*). In contrast, ERK1/2 activation by epidermal growth factor (EGF) was not affected by C1orf27 knockdown (Fig. S2). These data suggest that the normal function of C1orf27 is required for both the transport and function of α_2A_-AR.

### C1orf27 knockout by CRISPR-Cas9 attenuates the ER–Golgi-surface transport of α_2A_-AR

**Figure 5:**
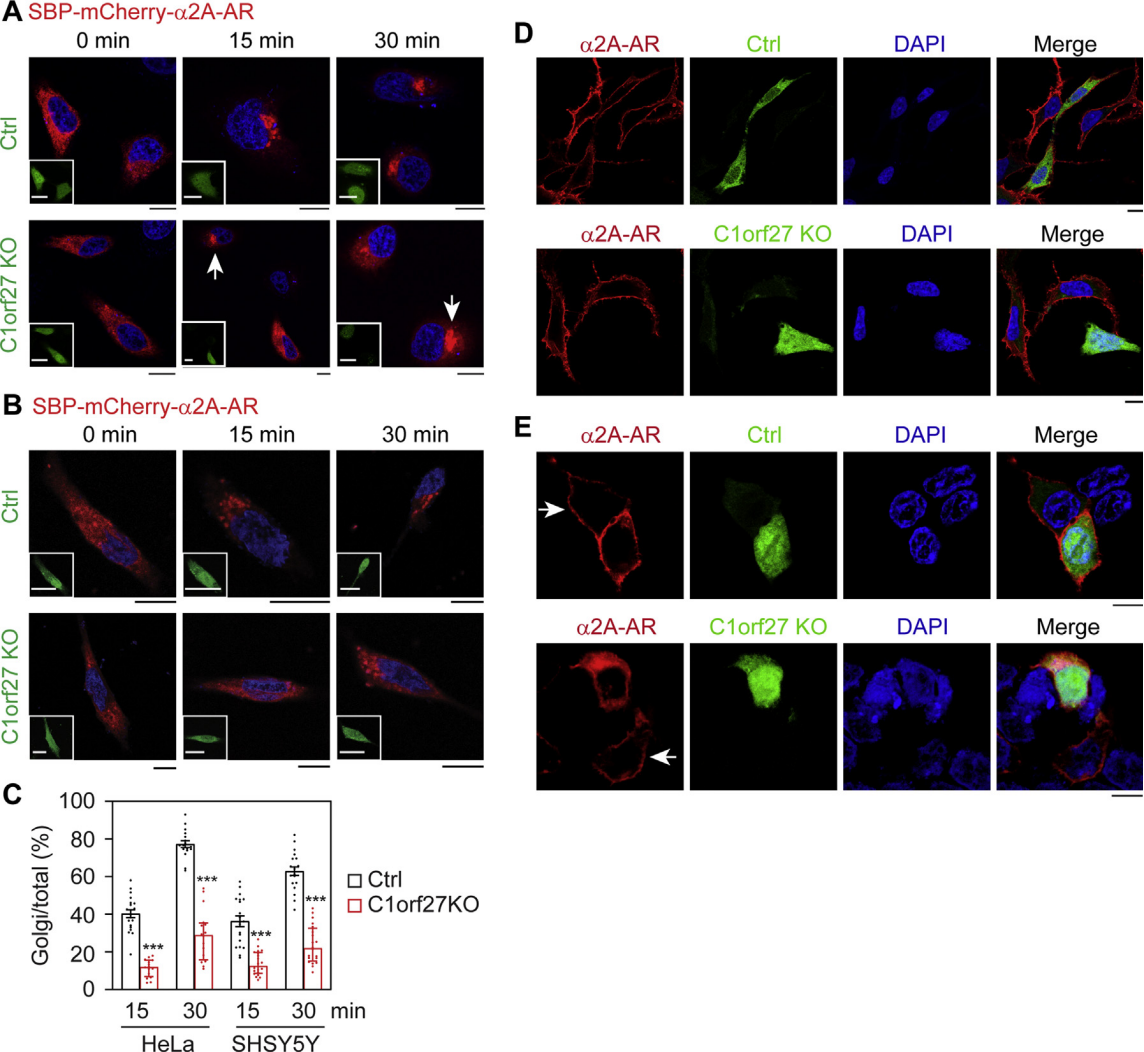
**CRISPR-Cas9–mediated C1orf27 KO suppresses α**_**2A**_**-AR export from the ER to the Golgi and the cell surface**. *A* and *B*, inhibition of ER–Golgi transport of α_2A_-AR in HeLa (*A*) and SHSY5Y cells (*B*) as measured in RUSH assays. The cells were transfected with Str-KDEL_SBP-mCherry-α_2A_-AR together with control (*upper panel*) or C1orf27 KO plasmids (*lower panel*) and fixed at 15 or 30 min after addition of biotin. Inserts show GFP expression. *Arrows* indicate cells without transfection of C1orf27 KO plasmids in which α_2A_-AR was exported to the Golgi. *C*, quantitative data shown in A and B. *D*, C1orf27 KO abolishes the surface transport of stably expressed α_2A_-AR. HEK293 cells stably expressing HA-α_2A_-AR were transfected with control or C1orf27 KO plasmids and stained with HA antibodies in nonpermeabilized cells. *E*, intracellular accumulation of α_2A_-AR in cells expressing C1orf27 KO plasmids in HEK293 cells. The cells were transfected with α_2A_-AR-RFP together control (*upper panel*) or C1orf27 KO plasmids (*lower panel*) carrying GFP. *Arrows* indicate cells without transfection with control plasmids (*upper panel*) or C1orf27 KO plasmids (*lower panel*) in which α_2A_-AR was expressed at the surface. The quantitative data shown are the Golgi/total expression ratio and expressed as mean ± SE (n = 16–20 cells in three separate experiments). ∗∗∗*p* < 0.001 *versus* control. Scale bars, 10 μm; Scale bars for inserts, 20 μm. α2A-AR, α2A-adrenergic receptor; ER, endoplasmic reticulum; GFP, green fluorescent protein; HA, hemagglutinin; KO, knockout; RFP, red fluorescent protein; RUSH, retention using the selective hooks.

To further confirm the role of C1orf27 in α_2A_-AR transport, we determined the effect of C1orf27 knockout (KO) by the CRISPR-Cas9 genome editing technology *via* transient transfection of KO plasmids. Because the control and KO plasmids carried GFP, the transfected cells were defined by the GFP signal. As α_2A_-AR functions mainly in neurons, human-derived neuroblastoma SHSY5Y cells were also used in these experiments. In the first experiment, the control or KO plasmids were transfected together with the RUSH plasmid Str-KDEL_SBP-mCherry-α_2A_-AR. In the absence of biotin, α_2A_-AR was expressed in the ER in cells transfected with control or C1orf27 KO plasmids. After induction with biotin for 15 or 30 min, α_2A_-AR expression at the Golgi was obvious in control cells expressing GFP or cells without transfection (indicated by *arrows*), whereas the receptor largely remained in the ER at 15 min and only partially exported to the Golgi at 30 min in C1orf27 KO cells (Fig. 5, *A–C*).

In the second experiment, we measured the effect of C1orf27 KO on the surface expression of α_2A_-AR in cells stably expressing HA-α_2A_-AR. After transfection with control or C1orf27 KO plasmids, the cell surface expression of α_2A_-AR was revealed by imaging after staining with HA antibodies in nonpermeabilized cells. As expected, α_2A_-AR was robustly expressed at the surface in cells transfected with control plasmids expressing GFP alone or without transfection, whereas the receptor was almost undetectable in cells expressing C1orf27 KO plasmids (Fig. 5*D*).

In the third experiment, we measured the effect of C1orf27 KO on the subcellular localization of α_2A_-AR after transient expression of α_2A_-AR-RFP. α_2A_-AR clearly expressed at the cell surface in cells transfected with control plasmids or without transfection (indicated by arrows), whereas the receptor was largely arrested in the perinuclear region, unable to transport to the surface, in cells transfected with C1orf27 KO plasmids defined by the GFP signal (Fig. 5*E*). These data further demonstrate a crucial role of C1orf27 in the ER–Golgi-surface traffic of α_2A_-AR.

### Effect of C1orf27 on the ER–Golgi-surface export of other GPCRs and epidermal growth factor receptor

**Figure 6:**
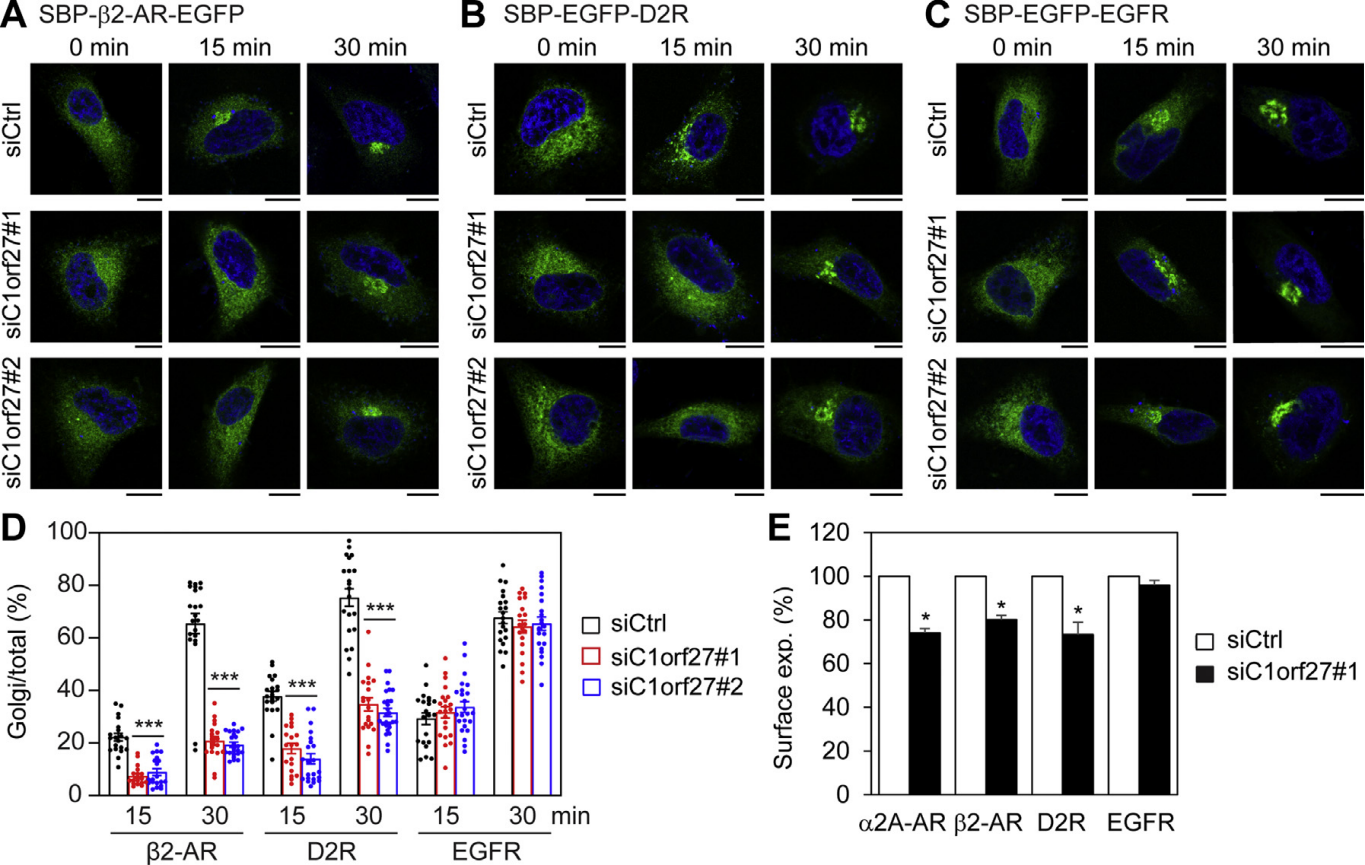
**C1orf27 depletion inhibits the export of β**_**2**_**-AR and D2R, but not EGFR, from the ER through the Golgi to the cell surface**. *A*–*C*, effect of C1orf27 knockdown by siRNA on the ER–Golgi transport of β_2_-AR (*A*), D2R (*B*), and EGFR (*C*) in RUSH assays in fixed cells. HeLa cells were transfected with individual receptor plasmids together with control or C1orf27 siRNA and fixed at 15 and 30 min after addition of biotin. Scale bars, 10 μm. *D*, quantitative data shown in *A–C*. *E*, effect of C1orf27 siRNA on the surface expression of α_2A_-AR, β_2_-AR, D2R, and EGFR as measured in BRET assays. The quantitative data are expressed as mean ± SE (n = 17–26 cells in three experiments in *D* and n = 3 in *E*). ∗*p* < 0.05 and ∗∗∗*p* < 0.001 *versus* control siRNA. α2A-AR, α2A-adrenergic receptor; β2-AR, β2-adrenergic receptor; BRET, bioluminescence resonance energy transfer; D2R, dopamine D2 receptor; EGF, epidermal growth factor; EGFR, EGF receptor; RUSH, retention using the selective hooks.

We next investigated if C1orf27 could regulate the export trafficking of other GPCRs and non-GPCR PM proteins. For this purpose, we measured the effect of siRNA-mediated knockdown of C1orf27 on the ER-to-Golgi trafficking of β_2_-AR, dopamine D2 receptor (D2R), and epidermal growth factor receptor (EGFR) in RUSH assays. Similar to α_2A_-AR, the ER-to-Golgi transport of both β_2_-AR and D2R at 15 and 30 min after addition of biotin was much less in C1orf27-depleted cells than in control cells (Fig. 6, *A* and *B*, *D*). In contrast, C1orf27 knockdown had no effect on the transport of EGFR (Fig. 6, *C* and *D*).

We then measured the effect of C1orf27 siRNA on the surface expression of α_2A_-AR, β_2_-AR, D2R, and EGFR at steady state after transient transfection in BRET assays. The surface expression of all three GPCRs, but not EGFR, was moderately but significantly attenuated by C1orf27 depletion (Fig. 6*E*). These data suggest that C1orf27 is likely a specific regulator for the transport of GPCRs.

### C1orf27 directly interacts with α_2A_-AR

**Figure 7:**
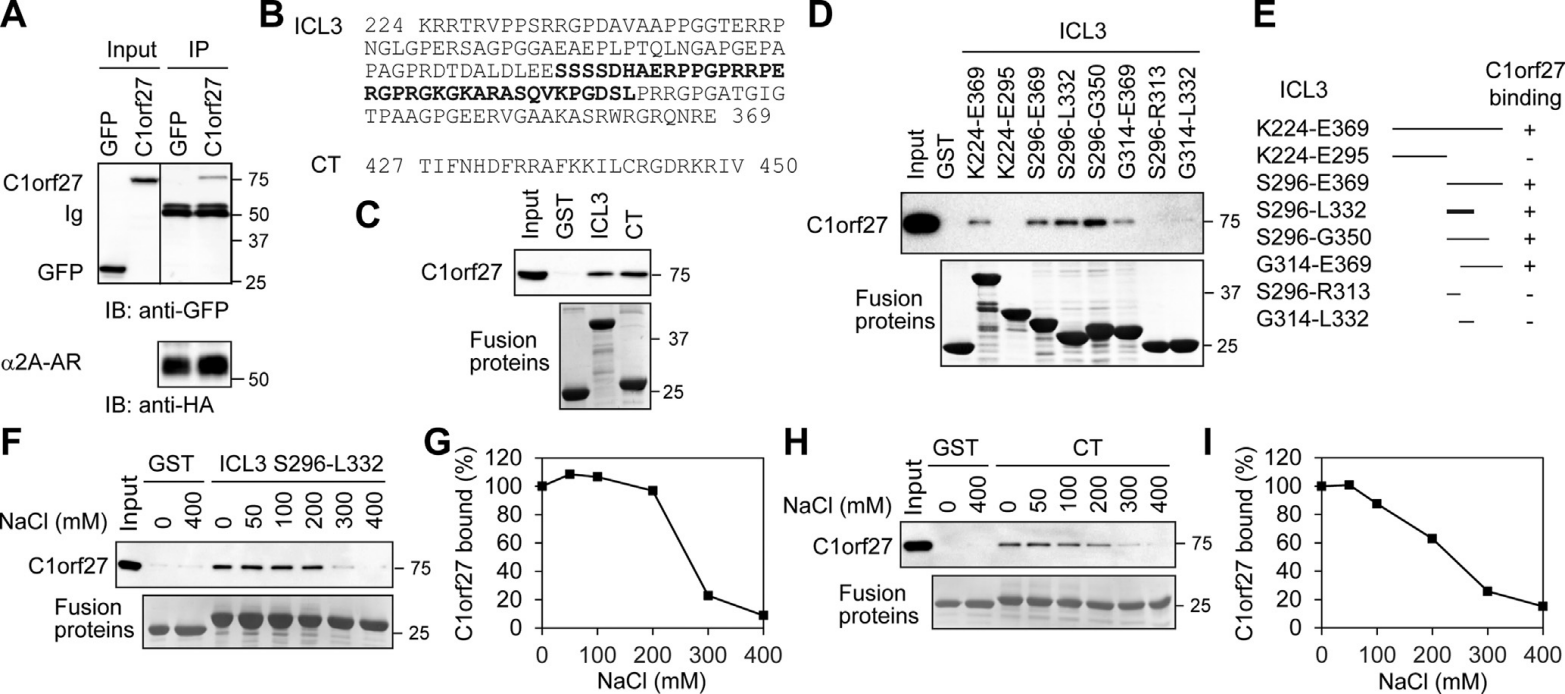
**C1orf27 interaction with α**_**2A**_**-AR and identification of the C1orf27-binding sites.***A*, co-IP of α_2A_-AR and C1orf27. HEK293 cells were transfected with HA-α_2A_-AR together with GFP-C1orf27 and subjected to IP with HA antibodies. *B*, sequences of the ICL3 and the CT of α_2A_-AR. The C1orf27-binding domain in the ICL3 as identified in D is *bolded*. *C*, interaction of the ICL3 and the CT of α_2A_-AR with C1orf27 in GST fusion protein pulldown assays. *D*, interactions of different ICL3 fragments with C1orf27 in GST fusion protein pulldown assays. *E*, summary of progressive deletion to identify the C1orf27-binding domain in the ICL3 of α_2A_-AR as shown in D. *F*, effect of increasing concentrations of NaCl on C1orf27 interaction with S296-L332. *G*, quantitative data shown in F. *H*, effect of increasing concentrations of NaCl on C1orf27 interaction with the CT. *I*, quantitative data shown in H. In each panel, similar results were obtained in at least three separate experiments. Lower panels in C, D, F, and H show GST fusion proteins used in individual experiments. α2A-AR, α2A-adrenergic receptor; Co-IP, co-immunoprecipitation; CT, C terminus; GFP, green fluorescent protein; GST, glutathione S-transferase; HA, hemagglutinin; ICL3, third intracellular loop.

To study the mechanisms underlying the function of C1orf27 in regulating α_2A_-AR transport, we determined if C1orf27 could interact with the receptor. In co-immunoprecipitation assays, C1orf27 tagged with GFP and α_2A_-AR tagged with hemagglutinin (HA) were co-expressed in HEK293 cells and were found to form a complex following immunoprecipitation with HA antibodies (Fig. 7*A*).

We next sought to identify the binding domains of α_2A_-AR in glutathione S-transferase (GST) fusion protein pulldown assays using the ICL3 and the CT of α_2A_-AR (Fig. 7*B*) which are known to mediate receptor interaction with a number of regulatory proteins involved in signaling and trafficking (17, 18, 19, 20, 21, 22). We found that C1orf27 similarly interacted with both the ICL3 and the CT (Fig. 7*C*). The progressive deletion strategy revealed that the shortest fragment-bound C1orf27 was S296-L332 in the ICL3, which further split abolished the interaction (Fig. 7, *D* and *E*).

As the fragment S296-L332 and the CT possess 10 and nine positively charged residues, respectively (Fig. 7*B*) and C1orf27 is an acidic protein with a calculated pI of 5.6, we determined the effect of increasing salt concentrations on their interactions. Incubation with NaCl inhibited the fragment S296-L332 interaction with C1orf27, and inhibition was in a dose-dependent fashion (Fig. 7, *F* and *G*). Similarly, the CT interaction with C1orf27 was also inhibited by increasing concentrations of NaCl (Fig. 7, *H* and *I*). These data suggest that the interactions between C1orf27 and α_2A_-AR are most likely ionic.

## Discussion

In this study, we have identified C1orf27 as an important regulator in the anterograde transport of GPCRs in mammalian cells. Although ODR4, a chemosensory neuron-specific protein in *C. elegans*, was first demonstrated to assist the localization of some ORs to olfactory cilia almost a quarter of century ago (24), it remains unknown if its human homolog C1orf27, a ubiquitously expressed protein, regulates protein trafficking in mammalian cells. To address this issue, in this paper, we have measured the effects of depleting endogenous C1orf27 by siRNA and CRISPR-Cas9 on the ER–Golgi transport kinetics, surface expression, subcellular localization, and signaling of three non-OR family A GPCRs in HeLa, HEK293 and SHSY5Y cells. There are several interesting points regarding the regulation of GPCR trafficking by C1orf27.

First, our studies have provided direct evidence indicating that C1orf27 controls α_2A_-AR transport from the ER to the Golgi apparatus. This became evident as C1orf27 depletion markedly slowed down the ER–Golgi transport of α_2A_-AR as measured in RUSH assays in both live and fixed cells. In particular, C1orf27 depletion delayed the half time of ER–Golgi transport of α_2A_-AR by greater than 65%. This function of C1orf27 is consistent with its localization in the ER (28).

Second, we have demonstrated that C1orf27 depletion significantly attenuates α_2A_-AR expression at the cell surface. As measured in live cell-based BRET assays using RUSH plasmids and ELISA assays, C1orf27 depletion by siRNA significantly attenuated the surface expression of α_2A_-AR at steady state. As revealed by confocal microscopic analysis of receptor subcellular localization in individual cells, α_2A_-AR was extensively accumulated in intracellular compartments and was almost completely unable to export to the cell surface in C1orf27 KO cells which were defined by the GFP signal. These data suggest that in addition to ER–Golgi traffic, C1orf27 can affect the steady state abundance of α_2A_-AR at the surface which is the functional destination.

Third, we have demonstrated that C1orf27 depletion produces similar inhibitory effects on α_2A_-AR transport in HEK293, HeLa, and SHSY5Y cells and that in addition to α_2A_-AR, C1orf27 also regulates the export of β_2_-AR and D2R, but not non-GPCR EGFR. These data, together with previous studies showing the role of ODR4 in OR transport in *C. elegans* (24, 25), imply a specific, conserved function of ODR4/C1orf27 family proteins in the biosynthesis and forward trafficking of GPCR family members.

Fourth, in parallel with the reduction in the cell surface α_2A_-AR expression, C1orf27 depletion suppressed receptor-mediated signaling measured as ERK1/2 activation. As C1orf27 siRNA does not affect EGF-mediated ERK1/2 activation, these data suggest that reduced ERK1/2 activation in response to α_2A_-AR stimulation in C1orf27-depleted cells is likely due to less surface receptor expression. As such, C1orf27 modulates not only the surface trafficking but also the function of α_2A_-AR.

Another important finding of this paper is the direct interaction between C1orf27 and α_2A_-AR. Our data have identified two C1orf27-binding domains located in the ICL3 and the CT of α_2A_-AR and revealed that C1orf27-α_2A_-AR interactions are most likely ionic in nature. These data, together with previous studies showing that *C. elegans* ODR4 and ODR10 form a complex (24, 25), strongly suggest that the actions of C1orf27 on α_2A_-AR traffic is likely specific. It is interesting to note that a number of proteins involved in GPCR biosynthesis are able to interact with the receptors they regulate (6). For example, we have recently found that α_2B_-AR interacts Sec24 isoforms (32) which are components of COPII vesicles that exclusively transport nascent cargoes from the ER, the small GTPases Rab43 (31, 33) and Rab26 (34) which regulate receptor transport from the ER and the Golgi, respectively, and GGAs (Golgi-localized, γ-adaptin ear domain homology, ADP ribosylation factor-binding proteins) involved in receptor post-Golgi transport (35, 36). The interaction between α_2A_-AR and C1orf27 provides another evidence indicating that GPCRs may physically interact with regulatory proteins to control their own anterograde trafficking.

Although our data presented in this paper have clearly demonstrated that C1orf27 interacts with α_2A_-AR and regulates its ER–Golgi-surface transport, the direct relationship between the interaction and α_2A_-AR trafficking and how the interaction controls α_2A_-AR export from the ER and subsequent transport to the Golgi and the cell surface are still elusive. As previously suggested for ODR4 in OR maturation in *C. elegans* (24, 25), C1orf27 may function as a specific chaperone and its interaction with α_2A_-AR facilitates receptor folding, recruitment onto COPII vesicles, ER export, and/or ER-Golgi traffic. It is also possible that C1orf27 and other regulatory proteins may form a multiprotein complex which further interacts with the receptor, generating a specialized transport machinery to drive receptor export from the ER and transport to the Golgi. In support of this possibility, C1orf27 has been shown to interact with ubiquitin-fold modifier 1-specific protease 2 (28) whose *C. elegans* homolog ODR8 also regulates OR transport in chemosensory neurons (24, 25).

It has been well described that intracellular trafficking of GPCRs is a crucial factor to fine tune the precise functions of the receptors in the right place at the right time. Over the past decades, most studies on GPCR trafficking have focused on events involved in internalization and recycling (37, 38, 39, 40); comparatively much less effort has been made to address the question of how targeted GPCR forward delivery is achieved. Emerging evidence from the studies in recent years suggest that export of GPCRs from the ER to the cell surface is regulatable, mediated through multiple pathways, and in a cell type– and receptor-specific manner. The most important progress toward the understanding of export trafficking of GPCRs is the identification of highly specific, conserved motifs embedded within the receptors that dictate receptor export from the ER and the Golgi (32, 41, 42, 43, 44, 45, 46, 47, 48, 49, 50) and a number of regulatory proteins that may stabilize receptor conformation, facilitate receptor maturation, and promote receptor delivery to the PM (51, 52, 53, 54, 55, 56, 57, 58, 59, 60). Our data presented in this paper have revealed the functional importance of C1orf27 in the ER–Golgi-surface transport of α_2A_-AR, providing important insights into regulation of nascent GPCR targeting to the functional destinations.

## Experimental procedures

### Materials

Antibodies against C1orf27 were purchased from Proteintech. Antibodies against GFP, phospho-ERK1/2, and β-actin were from Santa Cruz Biotechnology. Antibodies against ERK1/2 and rabbit host antibodies against HA epitope tag were from Cell Signaling Technology. Mouse host antibodies against HA were from Roche. Alexa Fluor 594-conjugated secondary antibodies, horse radish peroxidase–conjugated HA antibodies, 1-Step Ultra TMB-ELISA substrate solution, Lipofectamine 2000, D-biotin, cycloheximide (CHX), and dynabeads protein G were from Thermo Fisher Scientific. UK14304 was obtained from Sigma-Aldrich. MagneGST glutathione particles were from Promega. All other materials were obtained as described elsewhere (49, 61).

### Plasmids and constructions

α_2A_-AR tagged with GFP at its CT or HA at its NT was generated as described previously (33). α_2A_-AR tagged with RFP in the pTagRFP-N vector or Rluc8 in the pRluc8-N1 vector was generated by PCR using the primers (forward, 5′-GATCCTCGAGATGGGCTCCCTGCAGCCGGACG-3’ and reverse, 5′-GATCGGTACCGTCACGATCCGCTTCCTGTCCC-3′). The plasmids encoding Venus-tagged C terminal 25 amino acid residues of kRas4B, β_2_-AR-Rluc8, and D2R-Rluc8 were kindly provided by Nevin A. Lambert (Augusta University) as described (30). To generate the RUSH plasmid Str-KDEL_SBP-EGFP-α_2A_-AR, α_2A_-AR was first mutated to remove one Fsel restriction site by QuickChange site-directed mutagenesis using the primers (forward, ACGCTGGTGTGCCTGGCGGGGCTGCTCATGCTGCTC; reverse, GAGCAGCATGAGCAGCCCCGCCAGGCACACCAGCGT) without changing the encoded amino acid sequence and then amplified by PCR using the primers containing Fsel and XbaI restriction sites (forward, 5′-GATCGGCCGGCCAGGCTCCCTGCAGCCG-3’; reverse, 5′-GATCTCTAGATCACACGATCCGCTTCCTGTCCCCC-3′). The PCR product and the plasmid Str-KDEL_SBP-EGFP-Ecadherin (Addgene #65286) (29) were digested with Fsel and XbaI enzymes, purified, and then ligated. A similar strategy was used to generate the RUSH plasmid Str-KDEL_SBP-mCherry-α_2A_-AR by using Str-KDEL_SBP-mCherry-Ecadherin (Addgene #65287) (29). To generate the RUSH plasmid Str-KDEL_SBP- α_2A_-AR-Rluc8, α_2A_-AR-Rluc8 in the pRluc8-N1 vector was first mutated to remove one Sdal restriction site by using primers (forward, CACCCCTTACTCCCTACAAGTGACGCTGACGCTG; reverse, CAGCGTCAGCGTCACTTGTAGGGAGTAAGGGGTG) and then amplified by PCR using the primers containing Sdal and Xbal restriction sites (forward, 5′-GATCCCTGCAGGTATGGGCTCCCTGCAGCCG-3’; reverse, 5′-GATCTCTAGATTACTGCTCGTTCTTCAGCACGCG-3′) and subcloned into Str-KDEL_SBP-EGFP-Ecadherin after release of EGFP-Ecadherin by Sdal and Xbal. C1orf27 tagged with GFP at its NT in the pEGFP-C1 vector was generated by PCR using the primers (forward, 5′- CCGCTCGAGAAATGGGAAGAACCTACATTG-3’ and reverse, 5′-GGGGTACCCCCTAATCACTGAAGTAATG-3′). To generate the plasmid Str-KDEL_SBP-β_2_-AR-EGFP, β_2_-AR-EGFP (62) was amplified by PCR using primers (forward, 5′-GATCCCTGCAGGTATGGGGCAACCCGGGAAC-3’; reverse, 5′-GATCTCTAGATTACTTGTACAGCTCGTCCATGCCG-3′), and the PCR product was then subcloned into Str-KDEL_SBP-EGFP-Ecadherin after release of EGFP-Ecadherin by Sdal and Xbal. To generate the plasmid Str-KDEL_SBP-EGFP-D2R, D2R was amplified by PCR using primers (forward, 5′-GATCGGCCGGCCA ATGGATCCACTGAATC-3’; reverse, 5′-GATCTCTAGATTAGCAGTGGAGGATCTTC-3′) and D2R-Rluc8 as a template. To generate the RUSH plasmid Str-KDEL_SBP-EGFP-EGFR, EGFR lacking signal peptide was amplified by using primers (forward, 5′-GATCGGCCGGCCA CTGGAGGAAAAGAAAG-3’; reverse, 5′-GATCTCTAGATTATGCTCCAATAAATTCACTGC-3′) and EGFR-EGFP (Addgene #32751) as a template. Both products were digested with Fsel and Xbal and inserted into Str-KDEL_SBP-EGFP-Ecadherin after release of Ecadherin by Fsel and Xbal. To generate the plasmid EGFR-Rluc8, EGFP in EGFR-EGFP was replaced by Rluc8 using the BshTl and Notl restriction sites. GST fusion protein constructs coding the ICL3 of α_2A_-AR were generated in the pGEX-4T-1 vector as described previously (35). A similar strategy was used to generate the constructs coding the CT and different lengths of the ICL3 using the primers (CT: forward, 5′-GCTAGGATCCACCATCTTCAACCAC-3′ and reverse, 5′-GTGCCTCGAGTCACACGATCCG-3’; K224-E295: forward, 5′- ATGCGGATCCAAGCGTCGCACCC-3′ and reverse, 5′-ATGCCTCGAGTTACTCCTCCAGGTCCAGCGC-3’; S296-E369: forward, 5′- ATGCGGATCCAGCTCGTCTTCCGACCACG-3′ and reverse, 5′-ATGCCTCGAGTTACTCGCGGTTCTGC-3’; S296-L332: forward, 5′-ATGCGGATCCAGCTCGTCTTCCGACCACG-3′ and reverse, 5′- ATGCCTCGAGTTACAGGCTGTCGCCCGG-3’; S296-G350: forward, 5′- ATGCGGATCCAGCTCGTCTTCCGACCACG-3′ and reverse, 5′- ATGCCTCGAGTTACCCCGGCCCTGCAGC-3’; G314-E369: forward, 5′- ATGCGGATCCGGTCCCCGGGGCAAAGG-3′ and reverse, 5′-ATGCCTCGAGTTACTCGCGGTTCTGC-3’; S296-R313: forward, 5′-ATGCGGATCCAGCTCGTCTTCCGACCACG-3′ and reverse, 5′- ATGCCTCGAGTTAGCGCTCGGGTCTGCGGG-3’; G314-L332: forward, 5′- ATGCGGATCCGGTCCCCGGGGCAAAGG-3′ and reverse, 5′-ATGCCTCGAGTTACAGGCTGTCGCCCGG-3′). All constructs used in the present study were verified by nucleotide sequence analysis.

### Cell culture and transfection

HEK293 and HeLa cells were cultured in Dulbecco's modified Eagle's medium (DMEM) with 10% fetal bovine serum (FBS). SHSY5Y cells were cultured in F12/Minimum essential medium (V/V =1:1) with 10% FBS. Transient transfection of cells were carried out by using Lipofectamine 2000.

### Generation of cell lines stably expressing α_2A_-AR

HEK293 cell lines stably expressing HA-α_2A_-AR were generated as described for HA-α_2B_-AR (34). Briefly, HEK293 cells cultured on 6-well dishes were transfected with 2 μg of HA-α_2A_-AR in the pcDNA3.1(+) vector for 24 h using FuGENE HD transfection reagent according to the manufacturer’s protocol. The cells were split into four 100-mm dishes and selected with G418 at a concentration of 600 μg/ml for 2 weeks. Stable transfectants were isolated and grown in DMEM containing 300 μg/ml of G418. The cells stably expressing HA-α_2A_-AR were confirmed by immunoblotting using HA antibodies and radioligand binding using [^3^H]-RX821002.

### siRNA-mediated C1orf27 depletion

Two Stealth RNAi duplexes (siRNA) targeting human C1orf27 (targeting sequences are GGGUGCUGUGAAAUGCAGAGCUUAU and CCACAGCAGUAAACCCAAAGUUAAA), as well as negative control med GC duplex, were purchased from Thermo Fisher Scientific, and siRNA-mediated depletion was carried out as described previously (62). Briefly, cells were cultured on 6-well plates overnight and then transfected with 60 pmol of control or C1orf27 siRNA per well by using Lipofectamine 2000 for 24 h. The cells were transfected again with the same amount of siRNA together with 1 μg of α_2A_-AR per well for 6 h and split into 12-well plates for 20 to 24 h. Depletion of C1orf27 was confirmed by immunoblotting.

### CRISPR-Cas9–mediated C1orf27 KO

The CRISPR-Cas9 C1orf27 KO plasmids targeting human C1orf27, as well as control plasmids, were purchased from Santa Cruz Biotechnology, and the experiments were essentially carried out as described previously (31). The C1orf27 KO plasmid consists of a pool of three plasmids, each encoding the Cas9 nuclease and a target-specific 20 nt single guide RNA (sgRNA). Three sgRNA sequences in human C1orf27 are GTTGAAGTGTTCGTCACAAA, GTTGAAGTGTTCGTCACAAA and TACATACCTGTCCTATTAAA. To determine the effect of C1orf27 KO on the subcellular localization of α_2A_-AR, cells were cultured on 12-well dishes and transfected with C1orf27 KO plasmids plus α_2A_-AR-RFP (0.5 μg each) for 30 h before imaging. As control and C1orf27 KO plasmids carry GFP, transfected cells were defined by the GFP signal.

### Fluorescence microscopy

To measure the effect of C1orf27 depletion on α_2A_-AR transport in live cells, images were captured using LAS X software at an interval of 1 min with a 63x objective on a Leica Stellaris five confocal microscope equipped with an Okolab UNO stage top incubator. The cells with low receptor expression without aggregation were chosen to be studied. Receptor expression at the Golgi and the whole cell were quantified by measuring the fluorescence intensities. The Golgi area of individual cells was defined by the region with highly concentrated receptors after biotin induction. To measure the effect of C1orf27 depletion on the subcellular localization and ER export to the Golgi and the cell surface of α_2A_-AR in fixed cells, the cells were fixed with 4% paraformaldehyde for 15 min. To measure the surface expression of HA-α_2A_-AR in stable cell lines, the cells were fixed and blocked with 0.24% normal donkey serum for 1 h. The cells were then stained with HA antibodies overnight and Alexa Fluor 594-conjugated secondary antibodies for 1 h.

### RUSH assays

RUSH assays were essentially carried out as described (29). For live cell RUSH assays, HeLa cells grown on 35 mm Petri dishes with glass bottom were transfected with 1 μg of Str-KDEL_SBP-EGFP-α_2A_-AR with or without cotransfection with siRNA. After washing twice with Dulbecco′s phosphate buffered saline and addition of 1 ml of DMEM (no phenol red) containing 10% FBS, 1 ml of biotin (80 μM), dissolved in no phenol red DMEM with 10% FBS plus cycloheximide (CHX, 800 μg/ml) was added to induce receptor export. For fixed cell RUSH assays, cells were seeded on 12-well plates with coverslips overnight and transfected with 500 ng of Str-KDEL_SBP-EGFP-α_2A_-AR together with siRNA or Str-KDEL_SBP-mCherry-α_2A_-AR plus CRISPR-Cas9 KO plasmids for 20 h. The cells were incubated with biotin plus CHX for different time periods as indicated in each figure and then fixed. The data were expressed as the ratio of Golgi expression to the total expression. In some experiments (Fig. 2*C*), the Golgi/total ratio at each time point was subtracted from the ratio at time 0 and then normalized to the highest ratio which was defined as 100%.

### BRET assays

The live cell–based BRET assays were used to measure surface receptor expression in HEK293 cells as described previously (30, 31). Briefly, cells were cultured on 12-well plates and transfected with 250 ng of individual receptors tagged with Rluc8 at their C termini or Str-KDEL_SBP-α_2A_-AR-Rluc8 and 750 ng of Venus-kRas or pcDNA3.1 together with control siRNA or individual siRNA targeting C1orf27 with Lipofectamine 2000. The cells were transferred to 6-well plates and cultured for additional 20 to 24 h. The cells transfected with RUSH plasmids were incubated with biotin at 40 μM for 2 h. The cells were harvested and split onto black 96-well plates. After addition of coelenterazine h (5 mM), luminescence was immediately measured using a Mithras LB940 photon-counting plate reader (Berthold Technologies GmbH). The BRET signals were calculated by dividing the emission intensity at 520 to 545 nm by the emission intensity at 475 to 495 nm.

### ELISA

HEK293 cells cultured on 6-well plates were transfected with 1 μg of HA-α_2A_-AR together with control or C1orf27 siRNA and split into 24-well plates. After 24 h, the cells were fixed with 4% paraformaldehyde for 20 min, washed three times with cold Tris-buffer saline, blocked with 1% bovine serum albumin for 1 h, and incubated with horse radish peroxidase–conjugated HA antibodies at a dilution of 1:5000 for 1 h. After washing for three times, the cells were incubated with 200 μl of 1-Step Ultra TMB-ELISA substrate solution for 15 min. 100 μl solution from each well was transferred into 96-well plates, and 100 μl of H_2_SO_4_ (1 M) was added to stop the reaction. The absorbance at 450 nm was measured in a SpetraMax M2 microplate reader (Molecular Device).

### Measurement of ERK1/2 activation

Cells were cultured on 12-well plates and transfected with α_2A_-AR. After 24 h, the cells were starved for 12 h before stimulation with UK14304 at 1 μM for 5 min. Stimulation was terminated by addition of 100 μl SDS gel loading buffer. ERK1/2 activation was determined by measuring their phosphorylation by immunoblotting as described previously (62).

### Co-immunoprecipitation

Co-immunoprecipitation assays were carried out as described previously (33). Briefly, cells were cultured on 10-cm dishes and transfected with HA-α_2A_-AR together with GFP-C1orf27 constructs (10 μg each) for 24 h. The cells were harvested and lysed with 500 μl of lysis buffer containing 50 mM Tris-HCl (pH 7.4), 150 mM NaCl, 1% Nonidet P-40, 0.5% sodium deoxycholate, 0.1% SDS, and 1% protease inhibitors for 1 h. After centrifugation, the supernatants were incubated with 2 μg of HA antibodies (Roche, mouse host) overnight at 4 °C, followed by incubation with 30 μl of protein G dynabeads for 1 h at 4 °C. The beads were collected and washed three times with lysis buffer. Immunoprecipitated proteins were solubilized with SDS gel loading buffer and detected by immunoblotting.

### GST fusion protein pulldown assays

GST fusion protein pulldown assays were carried out using the MagneGST pull-down system (Promega) as described essentially (33, 34). Briefly, GST fusion proteins were expressed in bacteria and purified by using glutathione purification system. Purified fusion proteins were analyzed by Coomassie Brilliant blue staining following SDS-PAGE before experiments. GST fusion proteins tethered to the glutathione beads were either used immediately or stored at 4 °C for no longer than 2 days. To measure C1orf27 interaction, purified GST fusion proteins were incubated with HEK293 cell homogenates expressing GFP-C1orf27 in a total volume of 400 μl binding buffer containing 20 mM Tris-HCl (pH 7.4), 140 mM NaCl, 1% Nonidet P-40, and 10% glycerol overnight at 4 °C. After washing three times with binding buffer, the bound proteins were solubilized in SDS gel loading buffer and detected by immunoblotting using GFP antibodies.

### Statistical analysis

Statistical differences were analyzed by using one way ANOVA. All the data were expressed as mean ± SE. Significance levels are ∗*p* < 0.05, ∗∗*p* < 0.01, and ∗∗∗*p* < 0.001.

## Data availability

All data presented are available upon request from Guangyu Wu (guwu@augusta.edu)

## Supporting information

This article contains supporting information.

## Conflict of interest

The authors declare that they have no conflict of interest with the contents of this article.
