# Supplementary material for: Human C1orf27 protein interacts with α2A-adrenergic receptor and regulates its anterograde transport
Source: J Biol Chem. 2022 May 10;298(6):102021. doi: 10.1016/j.jbc.2022.102021 (PMC9168726; doi:10.1016/j.jbc.2022.102021)
Supplement: Supplemental Figures S1 and S2 [file mmc1.pdf]

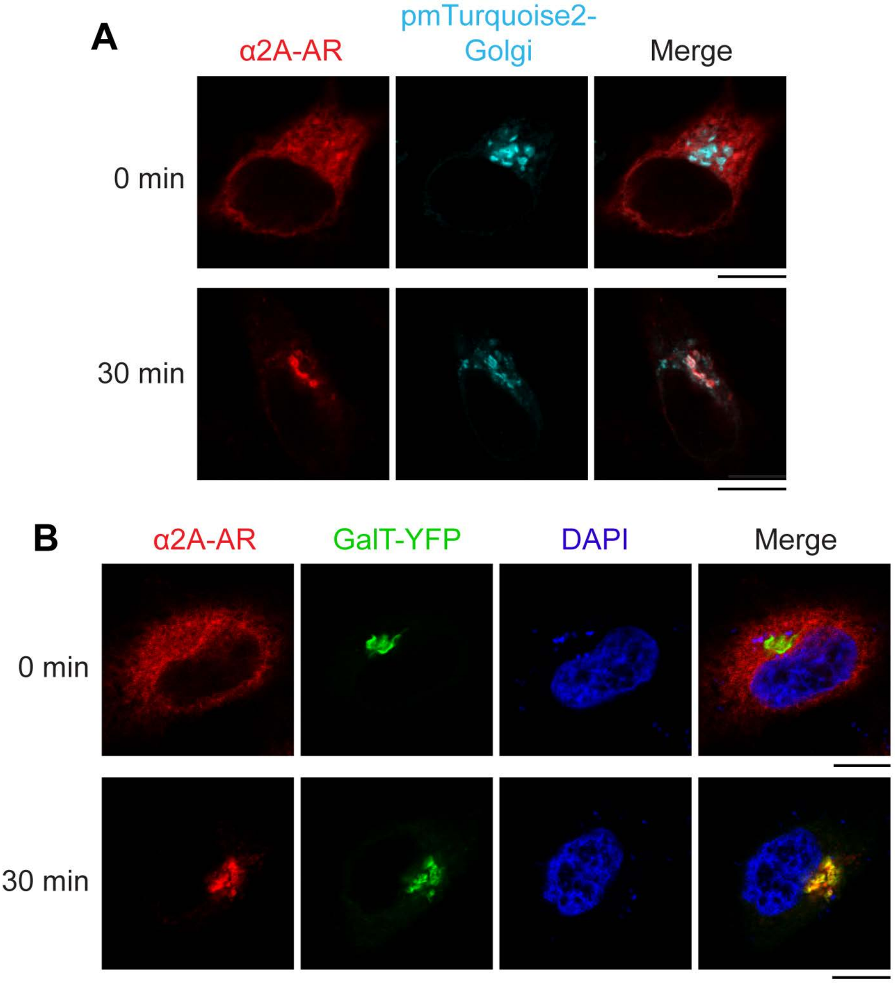

Fig. S1. Colocalization of α2A-AR with the Golgi markers. HeLa cells were transfected with Str-KDEL_SBP-mCherry-α2A-AR together with pmTurquoiase2-Golgi (A) or GalT-YFP (B) for 20 h and fixed at 30 min after addition of botin. Images shown are representatives of three individual experiments. Scale bars, 10 μm.

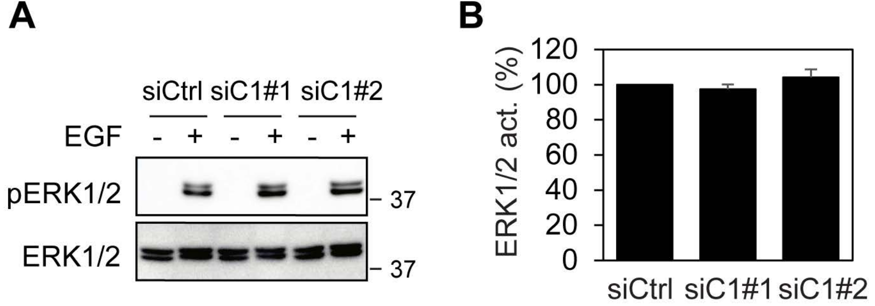

Fig. S2. Effect of siRNA-mediated knockodown of C1orf27 on ERK1/2 activatin by EGF.
A, Western blots showing that C1orf27 knockdown had no effect on ERK1/2 activation by EGF. HEK293 cells were transfected with control siRNA or siRNA targeting C1orf27 and stimulated by EGF at 50 ng/ml for 5 min. B, Quantitative data shown in A. The data are mean ± SE (n = 3).
